# POCUS to Confirm Intubation in a Trauma Setting

**DOI:** 10.5811/westjem.2020.9.49931

**Published:** 2021-01-20

**Authors:** Eric Scheier, Eli Shapiro, Uri Balla

**Affiliations:** * Kaplan Medical Center, Department of Pediatric Emergency Medicine, Rehovot, Israel; † Kaplan Medical Center, Department of Pediatric Critical Care, Rehovot, Israel; ‡ Faculty of Medicine, Hebrew University of Jerusalem, Israel

To the Editor:

In the recent edition of the *Western Journal of Emergency Medicine*, Gottlieb and colleagues discuss point of care ultrasound (POCUS) confirmation of intubation.^1^ Up to 25% of intubations using the classic formula of endotracheal tube (ETT) depth equal to three times the ETT diameter are inappropriately positioned,^2^ and 35–60% of mainstem intubations are missed by auscultation.^1^ Therefore, chest radiograph (CXR) has traditionally been used for confirmation of appropriate ETT placement.

In a prior review, Gottlieb reviews the utility of POCUS of the airway to demonstrate proper positioning of the ETT in real time.^3^ After publication of that review, Uya and colleagues identified the saline-filled ETT cuff on POCUS to demonstrate correct ETT depth in children undergoing cardiac catheterization.^4^ This technique builds on prior work suggesting that bilateral lung sliding can confirm appropriate placement of the ETT.^5^,^6^ However, bilateral lung sliding in adult studies is only 92–100% sensitive and 56–100% specific for confirmation of ETT position.^3^

To date, no authors have discussed the use of POCUS for confirmation of an appropriately placed ETT in a trauma setting in which the neck is obscured by a cervical spine collar. We wish to present a case which highlights the limitation in using bilateral lung sliding to confirm ETT placement.

A 12-year-old male was brought to our pediatric emergency department by emergency medical services. The child was intubated with a 6-0 uncuffed tube and his neck protected by cervical collar. The tube was secured with cloth tape. The tape and his cervical collar made it difficult to determine the ETT depth by inspection and difficult to perform POCUS of the trachea. POCUS using a modified Rapid Ultrasound for Shock and Hypotension (RUSH) protocol showed a bilateral “sand on the beach” pattern, indicative of pleural sliding in both hemithoraces, and the treating physician falsely assumed that the ETT was positioned correctly. The child’s initial CXR showed the tip of the ETT at the level of the carina. The ETT was unsecured and found to be at a depth of 21 centimeters at the lip. Repositioning to 18 centimeters at the lip resulted in appropriate positioning at the level of the sternoclavicular joints, as shown on repeat CXR.

Reliance on bilateral lung sliding to confirm appropriate ETT position is problematic. While absence of bilateral sliding may indicate a one-lung intubation, it cannot confirm a secured airway. In our experience, a low position of the ETT cuff is preferred to a high position, particularly in the prehospital setting where after intubation we are dealing with the transport of critically ill patients: a cuff position high in the trachea renders the ETT susceptible to dislodgement and risks loss of a definitive airway. In a child with bilateral lung sliding on POCUS, air in the ETT cuff, and a cervical collar, we wonder what information a transtracheal ultrasound adds. We question the safety of replacing air with saline in an established intubation in order to facilitate POCUS identification of a saline-filled cuff for confirmation of appropriate ETT placement.

We thank Gottlieb and colleagues for their important work. Further study is required to build a POCUS protocol to confirm an established intubation in a child with a cervical collar.
